# 3D Microstructures of Liquid Crystal Networks with Programmed Voxelated Director Fields

**DOI:** 10.1002/adma.202002753

**Published:** 2020-08-07

**Authors:** Yubing Guo, Hamed Shahsavan, Metin Sitti

**Affiliations:** Physical Intelligence Department, Max Planck Institute for Intelligent Systems, 70569, Stuttgart, Germany; Physical Intelligence Department, Max Planck Institute for Intelligent Systems, 70569, Stuttgart, Germany; Department of Chemical Engineering, University of Waterloo, Waterloo, ON N2L 3G1, Canada; Physical Intelligence Department, Max Planck Institute for Intelligent Systems, 70569, Stuttgart, Germany

**Keywords:** liquid crystal networks, molecular alignment, shape-change programming, soft robotics, two-photon polymerization

## Abstract

The shape-shifting behavior of liquid crystal networks (LCNs) and elastomers (LCEs) is a result of an interplay between their initial geometrical shape and their molecular alignment. For years, reliance on either one-step in situ or two-step film processing techniques has limited the shape-change transformations from 2D to 3D geometries. The combination of various fabrication techniques, alignment methods, and chemical formulations developed in recent years has introduced new opportunities to achieve 3D-to-3D shape-transformations in large scales, albeit the precise control of local molecular alignment in microscale 3D constructs remains a challenge. Here, the voxel-by-voxel encoding of nematic alignment in 3D microstructures of LCNs produced by two-photon polymerization using high-resolution topographical features is demonstrated. 3D LCN microstructures (suspended films, coils, and rings) with designable 2D and 3D director fields with a resolution of 5 μm are achieved. Different shape transformations of LCN microstructures with the same geometry but dissimilar molecular alignments upon actuation are elicited. This strategy offers higher freedom in the shape-change programming of 3D LCN microstructures and expands their applicability in emerging technologies, such as small-scale soft robots and devices and responsive surfaces.

Cross-linked networks of liquid crystal (LC) polymers possess reversible shape-shifting characteristics owing to the coupling of their rubber elasticity and anisotropic molecular order, or director field $\vec{n}$. When exposed to external stimulation, the temporary disruption of molecular order creates internal stresses leading to anisotropic bulk deformation.^[1,2]^ For decades, the shape-shifting behavior of these materials has been solely determined by the programming of their director field within a 2D initial geometry, such as a thin film. The majority of previous studies relied on the synthesis of liquid crystal elastomers (LCEs) based on renowned Finkelmann’s two-step technique of cross-linking and strain-induced alignment, which typically resulted in loosely cross-linked films with in-plane contraction upon heating (2D-to-2D transformation).^[3,4]^ Later, one-step polymerization with the in situ molecular alignment of reactive diacrylate mesogens introduced densely cross-linked LC networks (LCNs), which were able to demonstrate out-of-plane shape changes (2D-to-3D transformation).^[5,6]^ Advances in the control of molecular order in one-step film processing techniques, such as application of external fields^[7,8]^ and different surface alignment and photo-patterning techniques,^[9–22]^ led to various 2D-to-3D deformation profiles of varying degrees of complexity.

Shape programmability of both LCEs and LCNs has gained much attention in the burgeoning fields of soft robotics and stimuli-responsive structures and devices,^[23–37]^ albeit relying on simple 2D initial geometries, such as thin films, has hampered their extensive applications. In recent years, the development of new chemical formulations, like thiol–acrylate click chemistry, and novel fabrication techniques, like 3D printing, have introduced new opportunities in the shape-change programmability of LCEs and LCNs through the manipulation of their initial geometry to 3D shapes. Indeed, 3D-to-3D shape transformations have become viable either through simple molding, embossing, and selective photo-crosslinking of pre-polymerized LCE gels^[38–40]^ or by single- or multi-material 3D printing of LCE inks.^[41–45]^ Nevertheless, the molecular orientation in all these examples is controlled rather globally and mainly by the stretching and shear extrusion of the pre-polymerized LCE precursors.

Shape-change programmability of many soft robots and devices should also take account of the spatial addressing of desired components within a single construct, preferably without affecting other parts. To satisfy this requirement, Tabrizi and co-workers recently introduced a new technique for the voxel-by-voxel alignment of mesogens in a 3D LCE free form by combining a magnetic field for the alignment and a digital micromirror device for the selective photo-polymerization.^[46]^ The spatial control of the director field in LCE-based soft robots and devices becomes more challenging when the overall size of the construct goes down to the micrometer scale. Currently, soft-molding,^[25,47–49]^ traditional mask-based photolithography,^[50]^ and two-photon polymerization^[51–54]^ are the most common methods for the fabrication of 2.5D and 3D microstructures from LCEs and LCNs. However, the size of the molecular alignment domains in all reports, whether induced by magnetic fields or by the surface anchoring agents, has been equal to or larger than the overall size of the microstructures. As such, only simple uniform or twist director fields have been attained throughout these constructs.^[51–53]^

Here, we demonstrate 3D LCN microstructures with spatially controlled director fields obtained by two-photon polymerization of reactive mesogens that are locally oriented by surface topographical features. First, we show that microchannels fabricated from two-photon polymerization can be used for the 2D and 3D molecular alignment of LCN films adjacent to them with a resolution of ≈5 µm. Second, we achieve 3D LCN microstructures (e.g., coils and rings) by selective photo-polymerization of LCN precursor sandwiched between aligning microchannels. Such 3D LCN microstructures have spatially heterogeneous yet programmable director fields. Finally, we elicit different deformation profiles from 3D LCN microstructures with the same initial geometry but different encoded director fields. Microscale 3D LCE and LCN structures with programmable director fields are in high demand for their potential use in the development of soft microrobots and devices.^[24,55–60]^ We expect that our strategy will enable such advanced small-scale constructs with predetermined shape transformations necessary for their locomotion and functions.

To spatially control and encode the director field in 3D inside LCN microstructures, first, we need a robust microfabrication technique capable of creating well-defined 3D geometries from an LCN precursor. Second, we need to create local 3D alignment domains, or voxels, smaller than the whole size of the 3D construct with precisely encoded director fields. Currently, the two-photon polymerization is one of the most powerful techniques to create high-resolution 2D and 3D structures with sizes ranging from 200 nm to several millimeters. This technique has been effectively used for both the 3D fabrication of LCN microstructures with global molecular alignment^[51,52]^ and the high-resolution control of the molecular alignment of thermotropic and lyotropic LCs using microchannels and nano-pillars.^[61,62]^ We realize 3D LCN microstructures with spatially encoded director fields using two-photon polymerization by the fabrication of microchannels on the top and bottom glass substrates of a cell followed by the fabrication of 3D LCN micro-structures between them (Figure 1a). In such a design, micro-channels fabricated in the first step will be used to align the LCN director fields. This method works only if microchannels successfully generate the programmed director fields throughout pre-polymerized voxels without adverse interference from the neighboring counterparts. Moreover, the voxel-by-voxel cross-linking and freezing of nematic order during the two-photon polymerization of the designed 3D microstructure must not disturb the director field achieved in the first step. Throughout this study, we used a mixture of RM006:RM257 with a 2:1 weight ratio as the material of choice (Figure 1b). This material system has been widely studied and used in two-photon polymerization experiments.^[51–53,61,63]^ Details of the thermal and mechanical properties, and the order parameter of this material are elaborated in Figures S1–S3, Supporting Information.

We first demonstrate how microchannels confining our LC monomers, and LCNs thereof can generate patterns with a programmed director field. For this, we fabricated microchannels with 2 µm period and height on glass substrates by two-photon polymerization of a commercially available photoresist (IPS, Nanoscribe GmbH) using a direct laser writer (DLW, Nano-scribe GmbH). We fabricated two kinds of microchannels, one with continuous channels and the other with non-continuous pixelated channels (Figure 1c). Continuous channels with equal spacing are suitable for the fabrication of a limited number of special patterns such as circular +1 defect shown in Figure 1c(i). The pixelated design of microchannels with equal spacing, 10 µm × 10 µm pixels, overcomes this limitation and facilitates 2D, or in-plane, manipulation of the director field.^[62]^ Using such pixelated designs, we were able to fabricate a variety of patterns with microchannels, such as sinusoidal patterns with varying director field along a desired axis (Figure 1c(ii)), and a +2 topological defect (Figure 1c(iii)).

The alignment capability of continuous microchannels was examined using LC cells with identical +1 defects on the top and bottom substrates, as schematically shown in Figure 1d(i). The polarized optical microscope (POM) image of the pre-polymerized LCN mixture in Figure 1d(ii) indicates four bright regions around the defect center, which is a typical signature of a defect with a charge of +1, confirming the successful alignment of LCN monomers according to our design. We then polymerized the monomer mixture into an LCN film by flood exposure to ultraviolet (UV) light (Figure 1a(i)). Comparing the POM image after polymerization in Figure 1d(iii) to that of the pre-polymerized mixture in Figure 1d(ii), we can deduce that the molecular alignment is well-preserved even after polymerization. The director field is imperfect only within a 5 µm diameter in the vicinity of the defect center, signifying the high-resolution alignment of LCN.

To examine the alignment capability of pixelated arrays of microchannels, we used matching sinusoidal patterns on both sides of the LC cell (Figure 1e(i)). POM images of the pre-polymerized mixture and its polymerized counterpart are shown in Figures 1e(ii) and 1e(iii), respectively. Alternating bright and dark regions along the vertical direction confirm the effectiveness of our strategy in the alignment of pre-polymerized LC mixtures and LCNs thereof using pixelated matching patterns on both sides of the LC cell.

We extended the applicability of our method to create non-uniform director fields both in-plane and across the cell thickness, or a 3D director field and used LC cells with non-identical patterns on their opposing substrates. Note that throughout this work, we study only the twist nematic distortion along the thickness. Figure 1f(i),g(i) shows schematic cells with substrates of uniform alignment on the one side and substrates with +1 and +2 topological defects on the other, respectively. Corresponding POM images of pre-polymerized mixtures in Figure 1f(ii),g(ii) indicates typical two and four bright regions, respectively. Also, each bright region has a disclination in its center. Figure 1f(iii),g(iii) shows POM images with 45° rotation of the polarizers. After photo-polymerization, these 3D director fields are also well-preserved, as can be seen in Figure S4, Supporting Information. These results confirm the capability of our method in the 3D programming of the director fields using pixelated microchannels.

Due to the difference between the two-photon and flood UV polymerization, we need to optimize the parameters of the DLW to achieve well-defined 3D LCN microstructures with a programmed director field in each voxel. The fabrication procedures are very similar to the previous steps. However, as shown in Figure 1a(ii), we use two-photon polymerization of the LC mixture using DLW instead of flood exposure to UV (Figure 1a(i)) in this case. After the selective polymerization, we remove the top glass substrate and develop the remaining unpolymerized LC mixture with isopropanol. The key parameters affecting the fabrication procedure are the laser scanning speed, the laser intensity, and the development time. Higher laser scanning speed (solid scan speed = 10^5^ µm s^–1^) and shorter development time (3 min in isopropanol at elevated temperature) proved to be very important to achieve LCN microstructures with well-defined features replicating the initial geometrical design. The differences between the nature of light absorption in flood exposure polymerization and two-photon polymerization processes might lead to dissimilar polymerization kinetics, that is, polymerization extent and rate. In a series of swelling experiments on samples prepared with both techniques, we confirmed that all LCN microstructures are fully polymerized regardless of the scanning direction of the laser and final shape of the LCN microstructure. Details can be found in S3 and Figure S8, Supporting Information.

To confirm that the molecular alignment can be preserved even after the two-photon polymerization process, we designed and fabricated uniformly aligned and simple 3D microstructures, such as suspending films, rings, and coils as shown in Figure 2a–c, respectively. The accuracy of the fabrication process can be observed by comparing the design sketches and corresponding scanning electron microscope (SEM) images shown in Figures 2a(i–iii), b(i–iii), and c(i–iii). By comparing POM images with the director field along polarizers axis (Figure 2a(iv),b(iv),c(iv) and Figure S5, Supporting Information) and those with the director field 45° relative to polarizers axis (Figure 2a(v),b(v),c(v)), we observed that the designed uniform alignment throughout the fabricated 3D LCN microstructures is preserved after two-photon polymerization. We checked the grating effects from microchannels and found that surface gratings do not have an appreciable birefringence effect in our experiment (Figure S6, Supporting Information).

In pursuit of the voxelated encoding of the director field within a 3D LCN microstructure, we combined the molecular alignment technique using pixelated arrays of microchannels and two-photon polymerization. We started by the fabrication of LCN constructs with an in-plane non-uniform director field using the channels with the sinusoidal pattern previously demonstrated in Figure 1c(ii). For this, we used identical patterns on the top and bottom substrates. As shown in Figure 3a, the thin films (2D initial geometry) fabricated by DLW have a sinusoidal director field indicated by alternating bright and dark regions in POM images.

The fabrication of LCN microstructures with 3D initial geometry requires more scrutiny due to the importance of the correct positioning of the polymerization pathway on the aligning microchannels. The visibility of microchannels from the optical system of the DLW instrument simplifies this process and hints at the correct location and direction that LCN microstructures should be written. Figure 3b,c shows rings and coils fabricated with a sinusoidal director field, respectively. Coils show similar bright and dark regions consistent with the designed sinusoidal director field previously seen in 2D films. From the schematic in Figure 3c(i), we observe that the director field around the ring changes from ≈9° from the top to ≈45° in the middle and then back to ≈90° on the bottom, which results in two bright and black regions around the ring (Figure 3c(ii),(iii)).

We also used cells patterned with +1 topological defects on the top and bottom substrates for the fabrication of 2D films (Figure 3d), and 3D rings (Figure 3e). Corresponding POM images show four bright and black regions for both film and ring geometries, demonstrating the signature director field of +1 topological defect. Rings with the director field of +2 topological defects are also fabricated and presented in Figure 3f. The corresponding eight bright and dark regions around the coil under crossed polarized microscope indicate the successful fabrication and preservation of programmed molecular alignment (Figure 3f(ii),(iii)).

Next, we carried out the fabrication of 3D LCN microstructures with non-uniform director fields both in-plane and across the thickness using cells with mismatched patterned substrates. Again, we focused on twist nematic distortion of the director along the thickness direction. In the simplest scenario, we used substrates with uniform alignment on the top and bottom but with ≈80° angle between them. Note that liquid crystals tend to twist with a smaller angle for smaller distortion energy. Therefore, in most areas, liquid crystals twist from the bottom surface to the top surface with a fixed twisting sense. Only in some limited areas, the twisting is reversed, and these areas are surrounded by disclination loops. In the laser writing process, we can see and easily avoid these disclinations. In order to capture and examine the twisting nature of nematic order along with the thickness, we fabricated four suspending LCN films with similar thickness but at different heights from the bottom substrate (at 0–5, 6–11, 12–17 , and 18–23 µm). Assuming a linear change of the director field from the bottom substrate to the top one, we speculate that these four films have average director angles of around 85°, 73°, 61°, and 49°, respectively. Indeed, these films show different transmission intensity when observed under a cross-polarized microscope (Figure 4a(ii),(iii)).

A spiral coil is another good example of the optical examination of the twisting director field along the thickness (Figure 4b(i)). The top and bottom halves of the coil show two groups of oblique lines with different angles under a microscope. Since each coil is written from the bottom surface to the middle of a cell, there should be approximately 45° difference of director angle from bottom to the top of each coil. Under a cross-polarized microscope, the bottom half is invisible since the director field is closer to the optical axis of one polarizer (Figure 4b(ii)). After 35° rotation of the two polarizers, the top half of the coils becomes invisible. In a complex scenario, we introduced an in-plane variation of director fields on one side of the cell using our sinusoidal 2D patterns. For the coil schematically shown in Figure 4c(i), we observed that, in addition to the light intensity difference between the top half and bottom half, there exist alternating bright and dark regions due to the in-plane variation of the director field introduced by the microchannels on the top substrate (Figure 4c(ii)).

Finally, we fabricated rings with three different 3D director field configurations (Figure 4d–f). When the bottom substrate has the sinusoidal aligning pattern and the top substrate is uniformly aligned (Figure 4d(i)), the POM image (Figure 4d(ii)) shows two bright and dark regions similar to that in Figure 3c(ii). However, the dark regions disappear when polarizers are rotated by 45° (Figure 4d(iii)) as a result of the complex director field of ring compared with those shown in Figure 3c. When the director field on the bottom substrate is changed to +1 topological defect (Figure 4e(i)), we observed two dark regions and two disclinations in bright regions (Figure 4e(ii)) in POM image, resembling the POM image of LC monomer in Figure 1f(ii). Similarly, after the rotation of polarizers, all dark regions disappeared (Figure 4e(iii)). When the director field on the bottom substrate was changed into +2 topological defect (Figure 4f(i)), optical effects similar to that of Figure 1g(ii),(iii) were observed for Figure 4f(ii),(iii).

To induce and characterize the 3D-to-3D shape transformation of the fabricated 3D LCN microstructures, we performed a series of swelling experiments by dipping them into an isotropic solvent, such as N,N-dimethylformamide (DMF). Prior studies on the swelling behavior of LCEs and LCNs with isotropic solvents show that these materials experience larger swelling and deformation perpendicular to their local molecular orientation. Such anisotropic shape transformation is the result of different solvent uptake and the extent of order-disorder transition taking place parallel and perpendicular to the local director.^[64,65]^

Prior to our 3D-to-3D shape transformation experiments, we conducted a series of swelling tests to determine the swelling anisotropy. The swelling of uniformly aligned cubic samples of 60 µm in size in DMF resulted in ≈7% contraction parallel and ≈47% extension perpendicular to the local alignment (Figure S7, Supporting Information). Such anisotropic swelling in our samples can be utilized to render dramatic 3D shape transformations for the samples with a non-uniform director field.

The first example is the 3D-to-3D shape transformation of coils with similar initial geometrical features (150 µm length, 20 µm pitch, and 20 µm diameter), as shown in Figure 5a. Unlike their geometrical features, each coil had a different director field configuration as well as swelling behavior (Figure 5a(i)–(iv)). When the alignment was uniform throughout the coil, swelling in DMF resulted in pronounced expansion perpendicular to the director field (Figure 5a(i)). The shape transformation became complex for coils with a global twisted alignment, as they bent along their helical axes, in addition to expansion along their helical axis (Figure 5a(ii,iii)). Bending amplitude became larger in Figure 5a(iii) when the initial thickness of the LC cell decreased from 40 to 25 µm, shown by the 45° and 70° bending angles. Such a difference in the bending amplitude is the result of a larger twisting angle along the thickness of a cell with a smaller gap. Figure 5a(iv) shows the deformation of the coil with a uniform alignment on the bottom substrate and a sinusoidal pattern on the top substrate. The shape transformation for this configuration is significantly different and coils squeeze into crumpled structures due to their complex director field.

We conducted similar experiments on rings (50 µm outer radius and 30 µm inner radius) with various director field configuration, shown in Figure 5b(i)–(iv). The ring with global uniform alignment expanded perpendicular to the director field to a much greater extent (Figure 5b(i)). Both rings with global twisting alignment expanded and bent out-of-plane (Figure 5b(ii,iii)). The sample fabricated in the LC cell with an initial smaller gap (25 µm) had larger deformation due to the higher twist angle along the thickness. The ring in Figure 5b(iv) had a uniform alignment on the bottom substrate and a +2 topological defect director field on the top substrate. This sample expanded in all directions to a relatively similar extent.

The fabrication of the cross-linked networks of LC polymers with 3D initial geometries and with voxelated programming of the director fields has been challenging to achieve even at the macroscale. The only report on an LCE with a truly 3D initial geometry and voxelated director field is achieved by Tabrizi and co-workers through the simultaneous use of rotating permanent magnets for the alignment and a digital micromirror device for the selective photo-polymerization of voxels.^[46]^ A relatively similar concept was used by Yao and co-workers for the magnetic alignment of mesogens to create LCE micro-structures with precisely encoded molecular order to achieve 3D shape transformation.^[49]^ However, the initial shapes in that work are merely limited to 2.5D geometries fabricated in soft-lithography procedures. Pioneering works by Zeng and co-workers undoubtedly paved the way for a better understanding of challenges and potential solutions for the voxelated programming of the director field at the micrometer scale.^[51,52,61]^ In a recent work, they demonstrated microrings with uniform alignment and microstripes with twisting alignment by using microchannels and two-photon polymerization.^[61]^ However, in this work, we have obtained much complex 3D structures with smaller feature sizes (e.g., coils) and higher resolution for the molecular alignment through our pixelated strategy. Moreover, we have improved the achievable modes of deformation of microstructured LCNs by varying the director field in different portions inside a single structure (voxel). In general, we have alleviated some of the shortcomings of the previous reports with regards to both the 3D molecular alignment and 3D fabrication by taking advantage of the two-photon polymerization technique.

With regard to the alignment, pixelated arrays of microchannels can provide a variety of 2D and twisting 3D patterns of the director field with high spatial resolutions down to ≈5 µm, as estimated from the misaligned region in the core of circular +1 defect pattern (Figure 3d) and distinct light intensities of suspended films with 5 µm thickness (Figure 4a).^[66]^ As shown by Yao and co-workers, the magnetic susceptibility of the LC molecules also creates an opportunity for the molecular alignment in a relatively programmable fashion. Nonetheless, its contribution in defining the spatial resolution of the molecular alignment is limited due to the larger contribution of surface anchoring when one of the dimensions approach to a critical length. This critical dimension can be determined by a balance between free energy induced by the local magnetic field and that induced by the surface anchoring using $\xi =\frac{W\mu_{0}}{\Delta \chi B^{2}}$. In this correlation, *W* is the surface anchoring strength, Δ*χ* is the diamagnetic anisotropy, *μ*_0_ is the free space permeability, and *B* is the strength of the local magnetic field. As such, the smallest dimension reported in Yao’s work was 25 µm, well above the resolution reported here.^[49]^ The resolution in our strategy solely depends on the surface anchoring strength, which can be readily manipulated by the alteration of surface topography and chemistry. In addition to the magnetic alignment, photoalignment techniques like plasmonic photo-patterning can provide high spatial resolution down to sub-micrometer. However, the transparency of the photoalignment patterns necessitates so-called alignment marks on substrates for the accurate positioning of the 3D microstructures to be written by DLW. Indeed, besides high spatial resolution, another advantage of using microchannels is that they are visible in a DLW system, which simplifies the positioning procedure of the 3D designs before the selective polymerization.

The distortion of the director field along the cell thickness crucially depends on the geometry, topography, and chemical properties of the top and bottom confining substrates. In this work, using flat substrates and in-plane arrays of microchannels for the molecular alignment limited the mode of nematic order distortion along the thickness to only twisting mode. Also, the height of achievable 3D microstructure is limited to ≈100 µm in order to obtain good alignment of the liquid crystal director field. However, we predict that these shortcomings can be addressed in the future by the application of multi-level or non-flat surface topographical features to induce local splay and bend types of nematic order distortions.

With regard to the fabrication, two-photon polymerization can be utilized in the creation of 3D LCN microstructures with high levels of geometrical complexity and liberty in shape programming. This method also allows a single 3D LCN microstructure to demonstrate multiple modes of shape transformation by careful design of its local director field. Furthermore, the modification of LC-based photoresists can diversify the repertoire of achievable mechanical properties, stimulation techniques, and functionality of LC-based 3D microstructures. Moreover, the modification of DLW setup itself with auxiliary apparatus, such as magnets for the molecular alignment and integrated microfluidic systems for multi-material fabrication,^[67]^ could offer new opportunities for the fabrication of multi-material and multi-functional 3D microdevices.

To summarize, we have demonstrated a strategy to fabricate voxelated 3D LCN microstructures with encoded 2D and 3D director fields. We first showed that pixelated arrays of microchannels can be used to introduce high-resolution encoding of director fields with designable 2D and 3D patterns in pre-polymerized LCNs. By combining this method with two-photon polymerization, we realized 3D LCN microstructures with voxelated designable patterns of director fields. Finally, we examined various 3D-to-3D shape transformation behaviors of the fabricated microstructures with similar initial shapes (either coils or rings), but different designed director fields. Our new strategy significantly expands the freedom in the design and fabrication of 3D LC-based microstructures and paves the way for their broader applications in many emerging technologies, such as small-scale soft robots and devices and stimuli-responsive surfaces.

## Supplementary Material

Supplementary material

Video S1

Video S2

Video S3

## Figures and Tables

**Figure 1:**
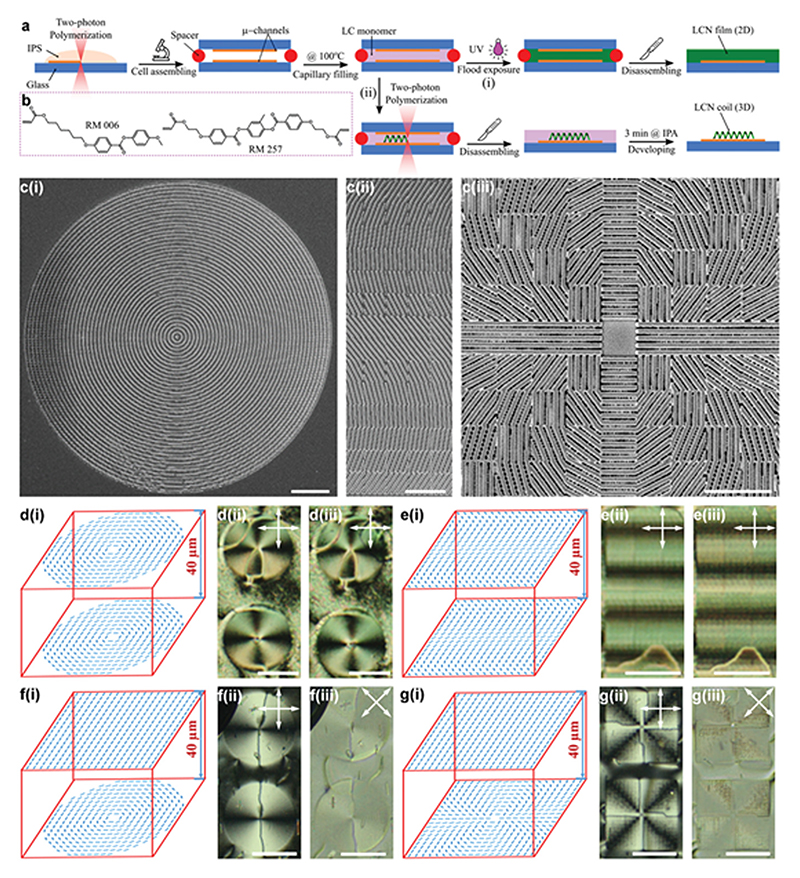
3D director field alignment of liquid crystal (LC) monomers. a) Schematic of the fabrication procedure and steps of 2D LCN films (i) and 3D LCN microstructures (ii). b) Chemical structures of LC monomers used: RM006 (left) and RM257 (right). c) Scanning electron microscopy (SEM) images of the microchannels fabricated by two-photon polymerization for a +1 defect pattern (i), a sinusoidal pattern (ii), and a +2 defect pattern (iii). d,e) Alignment of the LC monomer with a +1 defect pattern (d), and a sinusoidal pattern (e); (i), (ii), and (iii) show the schematic of the LC cells and the alignment pattern with given microchannel patterns, polarized optical microscope (POM) images of liquid crystal monomers, and POM images of LCN films, respectively. f,g) The alignment of LC monomers with a +1 defect pattern (f), and a +2 defect pattern (g) on the bottom substrate and uniform alignment on the top one; (i)–(iii) show the schematic of LC cells and director fields, POM images of LC monomers, and their POM images with rotated polarizers, respectively. Double-sided arrows exhibit the optical axis of the polarizers. Scale bars are 20 µm in (c) and 100 µm in (d–g).

**Figure 2:**
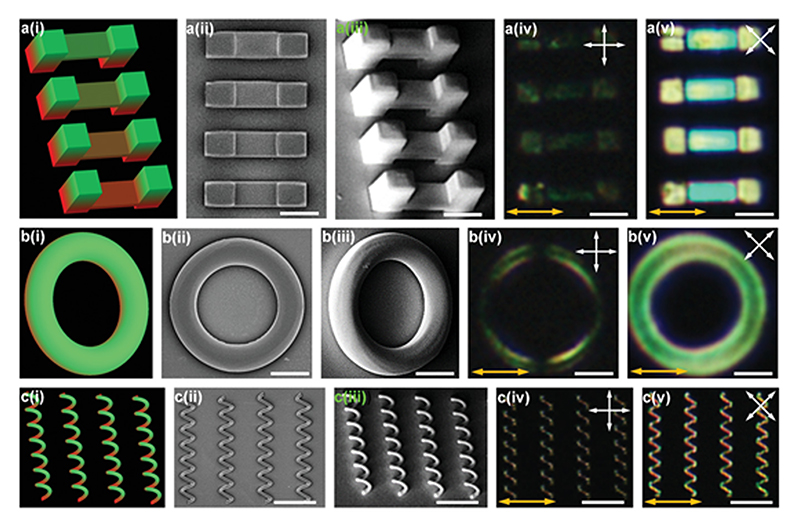
Fabricated 3D LCN microstructures with uniform director field alignment. a–c) Suspended films at different heights (a), rings (b), and spiral coils (c); (i)–(v) represent the designed microstructures (i), the top-view SEM image (ii), the tilted SEM image (iii), the POM image (iv), and the POM image with rotated polarizers (v), respectively. Double-sided arrows with white color exhibit optical axis of polarizers and ones with orange color show the liquid crystal director. Scale bars are 30 µm in (a,b) and 60 µm in (c). Note that in order to show the position of the fabricated structures in a(iv), b(iv), and c(iv), we increased the brightness of the POM images. The original images are shown in Figure S5, Supporting Information.

**Figure 3:**
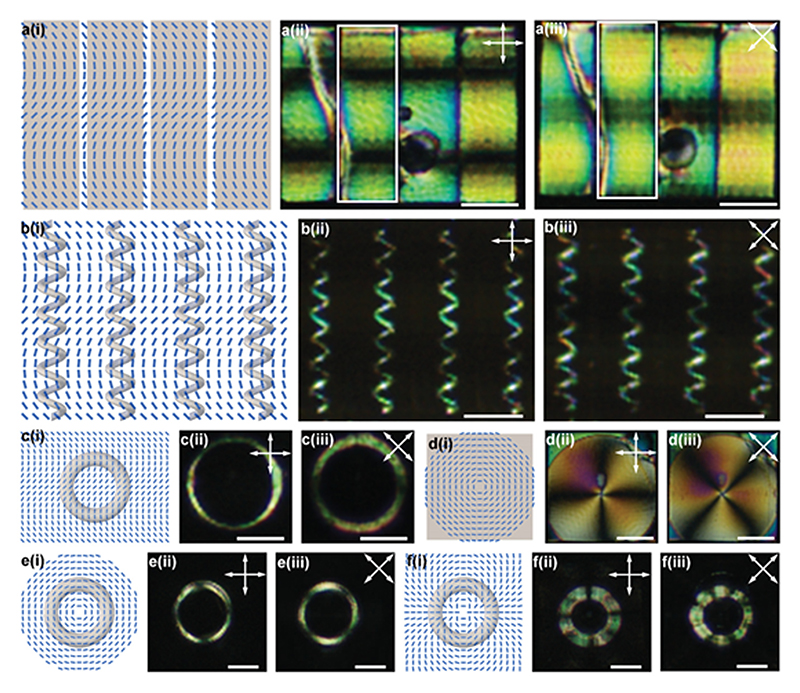
3D LCN microstructures with encoded 2D director field. a–f) Films with sinusoidal director field (a), coils with sinusoidal pattern (b), a ring with sinusoidal pattern (c), a film with +1 defect pattern (d), a ring with +1 defect pattern (e), and a ring with +2 defect pattern (f); (i), (ii), and (iii) show the schematic of director fields, POM images of LCN films and structures, and their POM images with rotated polarizers, respectively. Double-sided arrows exhibit the optical axis of the polarizers. All scale bars are 50 µm.

**Figure 4:**
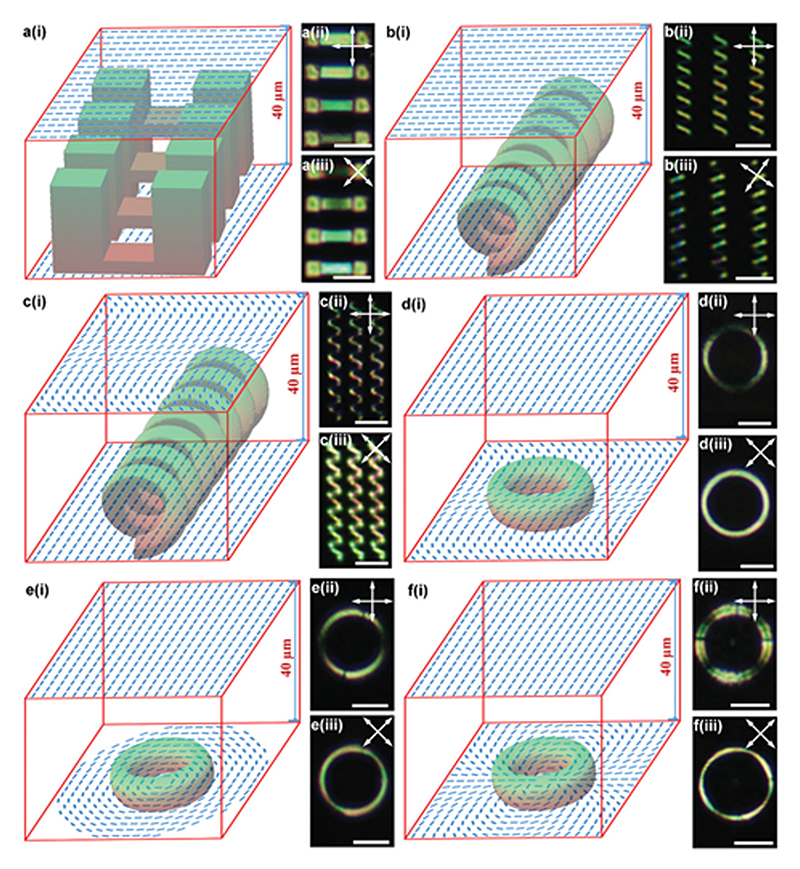
3D LCN microstructures with encoded 3D director field. a–f) Suspended films at different heights with twisting molecular alignment (a), coils with twisting molecular alignment (b), coils with a sinusoidal director field on top and uniform molecular alignment on the bottom (c), a ring with uniform alignment on top and sinusoidal pattern on bottom (d), a ring with the uniform molecular alignment on top and +1 defect pattern on bottom (e), and ring with uniform alignment on top and +2 defect pattern on bottom (f); (i), (ii), and (iii) represent the schematic of LC cells and director fields, POM images of LCN structures, and their POM images with rotated polarizers, respectively. Double-sided arrows exhibit the optical axis of the polarizers. All scale bars are 50 µm.

**Figure 5:**
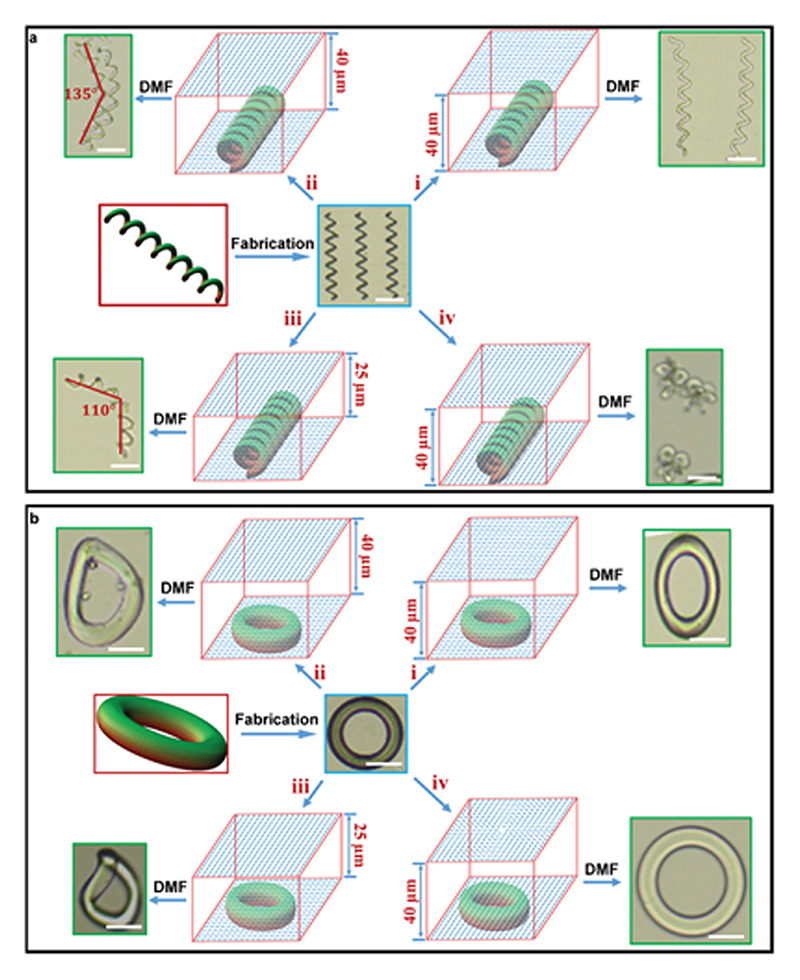
Various modes of 3D-to-3D shape transformation from LCN microstructures of the same geometry (coils and rings) but with different encoded 3D director fields. a) The shape transformation of coils: (i), (ii), (iii), and (iv) represent uniform alignment, twisting alignment with 40 µm thickness, twisting alignment with 25 µm thickness, and 3D alignment with a sinusoidal director field on the top substrate, respectively. Coils expanded perpendicular to the director field (i), expanded and bent with different amplitudes (ii,iii), and squeezed into crumpled structures (iv). b) The shape transformation of rings: (i), (ii), (iii), and (iv) represent uniform alignment, twisting alignment with 40 µm thickness, twisting alignment with 25 µm thickness, and 3D alignment with a +2 topological defect on the top substrate, respectively. The rings expanded perpendicular to the director field (i), expanded and bent out-of-plane with different amplitudes (ii,iii), and expanded uniformly in all directions (iv). Scale bars are 50 µm in all images. All shape deformations are achieved by swelling the LCN microstructures with *N,N*-dimethylformamide.
