# Supplementary material for: 3D Microstructures of Liquid Crystal Networks with Programmed Voxelated Director Fields
Source: Adv Mater. Author manuscript; Available in PMC 2021 Mar 29. (PMC7610484; doi:10.1002/adma.202002753)
Supplement: Supplementary material [file EMS104393-supplement-Supplementary_material.pdf]

© 2020 Wiley-VCH GmbH

ADVANCED MATERIALS

Supporting Information

for *Adv. Mater.*, DOI: 10.1002/adma.202002753

3D Microstructures of Liquid Crystal Networks with Programmed Voxelated Director Fields

*Yubing Guo, Hamed Shahsavan, and Metin Sitti**

Supporting Information

3D Microstructures of Liquid Crystal Networks with Programmed Voxelated Director Fields

*Yubing Guo, Hamed Shahsavan, Metin Sitti**

Dr. Y. Guo, Prof. H. Shahsavan, Prof. M. Sitti
Physical Intelligence Department, Max Planck Institute for Intelligent Systems, 70569 Stuttgart, Germany

Prof. H. Shahsavan
Department of Chemical Engineering, University of Waterloo N2L 3G1, Waterloo, ON, Canada

* E-mail: sitti@is.mpg.de

S1. Materials and Methods

Materials. The LC mixtures used in this work are based on different mass ratios of RM006 (Synthon Chemicals) and RM257 (Synthon Chemicals). The preliminary studies were conducted to measure the thermal properties of a variety of LC mixtures (RM006: RM257) with 1:3, 1:1, and 2:1 mass ratio. We chose RM006: RM257 mixture with 2:1 mass ratio as the main composition to produce LCNs, as shown in Figure 1b. We mixed 1 wt% of photoinitiator Irgacure-369 (Ciba) as the photoinitiator to the mixture. The LCN precursor mixture was mixed at molten state on a hot stage at 120 ^{o}C for 1 hr. Afterward, it was injected into cells with ~ 40 µm thickness at 100 ^{o}C, above its clearing point, to avoid flow alignment [The Journal of Chemical Physics 1966, 45, 1452−1456]. The liquid crystal cells were then cooled down to room temperature at the rate of 5 ^{o}C/hour for further procedures. Note that the very low cooling rate is crucial to reduce the number of disclinations especially for the complex director fields. In the last step, LCNs were synthesized by the photo-polymerization of the LC mixture either by UV flood exposure ($1 \mathrm{J/cm^2}$ 365nm UV light) or two-photon polymerization.

Laser writing of micro-channels. We fabricated micro-channels for the alignment of LCNs with a direct laser writing system (Nanoscribe GmbH) equipped with a 63X oil-immersion objective (NA=1.4). Negative photoresist IP-S 780 (Nanoscribe GmbH) was dispensed on a cleaned glass, and micro-channels with designed orientations were written into the IP-S 780 during the two-photon laser writing process. Then, the glass slides were dipped in propylene glycol monomethyl ether acetate (PGMEA) for 30 minutes to remove the unexposed photoresist.

Liquid crystal cells. For the 2D alignment of LCNs, we fabricated mirror micro-channel patterns on two glass substrates. The two substrates were then assembled with micro-channel patterns on two inner sides. UV glue mixed with spacers of the desired size was applied to the four corners of assembled substrates. Then two mirror patterns on two substrates were carefully positioned and aligned under a microscope. In order to align the two patterns well, we fixed the bottom substrate and adjusted the top substrate with translation and rotation, which was followed by UV curing of the glue to preserve the well-aligned patterns. Other kinds of liquid crystal cells are fabricated with a similar method without the alignment procedure under a microscope.

Laser writing of LCN micro-channels. We used a direct laser writing system (Nanoscribe GmbH) equipped with a 63X oil-immersion objective (NA=1.4) to create LCN micro-channels. The targeting area was found under the visual system of the laser writing system and then designed patterns were written with 780 nm laser at 45 mW power. Coils and rings with size in this paper took around 30 seconds and 120 seconds, respectively. Then, the cell was separated and the glass substrates with LCN microstructures were dipped in isopropanol at elevated temperature for three minutes to remove the unexposed photoresist.

S2. Material Characterization

Tensile tests. We performed the mechanical tensile test to determine the Young's modulus of the LCN films created by flood exposure photo-polymerization with 1:2 wt. ratio of RM257 to RM006 using a rheometer (TA Instruments, Discovery HB 20). We performed traditional stress-strain tests at room temperature on stripes of LCN (5 mm × 30 mm × 60 µm) with a

strain rate of 10 μm/s. Prior to the tensile experiments, the LCN stripes were cut in three directions: parallel to the director field, perpendicular to the director field, and with 45° angle with respect to the director field. Young's moduli of the samples were measured by calculating the slope of the stress-strain curves shown in Figure S1 within the 1% strain region. As expected, Young's modulus of our LCN is the highest when the direction of stress is parallel to the director field (~ 0.89 ± 0.006 GPa), and it decreases as the direction of stress changes to 45° (~ 0.740 ± 0.002 GPa), and 90° (~ 0.65 ± 0.004 GPa) with respect to the director field.

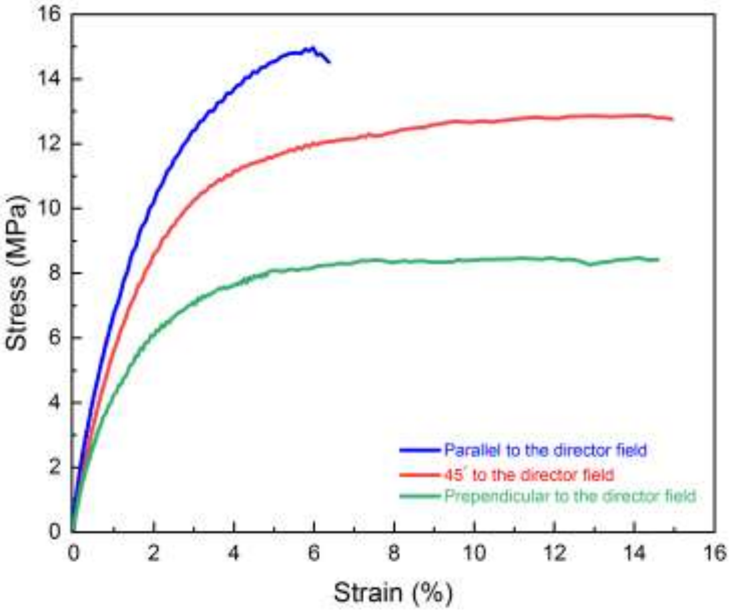

Figure S1. Stress-strain curves obtained during the tensile mechanical testing of LCN stripes cut parallel to, perpendicular to and with 45° angle to the director field.

Differential Scanning Calorimetry (DSC). In order to measure the glass transition temperature (Tg) of the LCN with 1:2 weight ratio of RM257 to RM006, and the nematic-to-isotropic temperature (T_{NI}) of pre-polymerized mixtures of RM257 and RM006, we monitored the variation of heat flux with temperature. The samples were cooled down to -40 °C and then heated up to 120 °C in two consecutive cycles (10 °C/min). Figure S2 shows the results of the heating in the second thermal cycle after erasing the thermal memory of the samples in the first cycle. Glass transition temperature of LCN was measured as ~ 29 °C. The evolution of heat flux with temperature for our LCN does not show a pronounced nematic-to-isotropic transition point.

It is worth noting that the higher concentrations of RM257 (cross-linker) result in pre-polymerized mixtures of remarkably higher T_{NI}. Also, the pre-polymerized mixture with higher RM257 and T_{NI} had shorter and more unstable periods in a defect-free nematic phase at room temperature. The process of two-photon polymerization takes place at room temperature and from mixing the monomers, to injection of the pre-polymerized mixture, and the eventual two-photon polymerization process usually requires hours of operation. We noticed that samples with higher concentrations of RM257 tend to experience some extent of crystallization, which acts as a potential source of defects in the pre-polymerized nematic mixture. Also, it is known that polymerization of LC mixtures of higher T_{NI} at room temperature may endure some degrees of vitrification, which limits monomers' mobility and further reaction of the unreacted groups [Langmuir 2014, 30, 13499−13509]. All these parameters led us to the 2:1 weight ratio of RM006 and RM257.

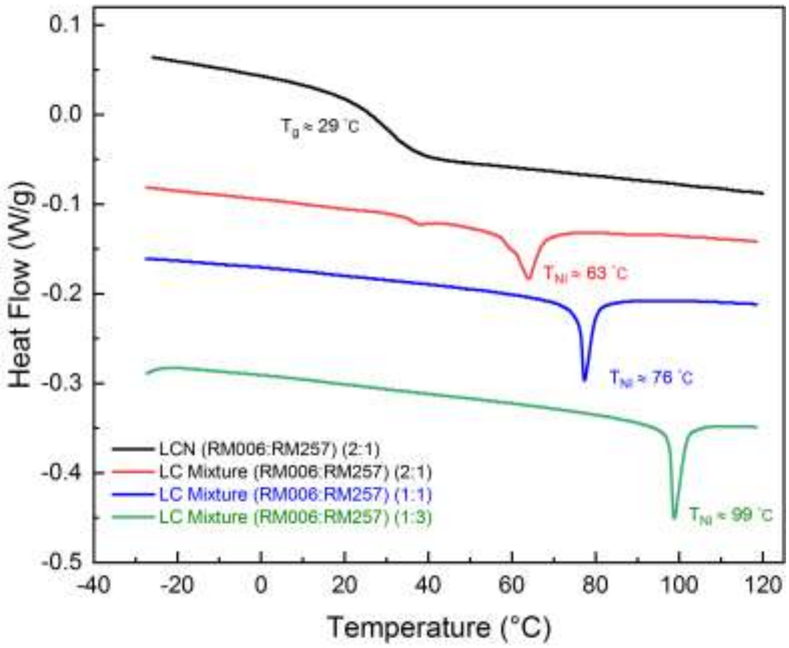

Figure S2. DSC characterization results showing the variation of heat flux with temperature for different samples.

Order Parameter. We measured the dichroism of the disperse red 1 dye, which was doped in LCN sandwiched in planar cells with 5 μm thickness, to have an approximation of the order parameter. Cells without dyes were also prepared as reference samples. Transmission spectra were measured with a UV-Vis plate reader (Synergy HTX Multi-Mode, Biotek). A broadband light was shone on samples through a linear polarizer, once with along the director field, and once perpendicular to it. We recorded the absorption spectra of dyes along the director field ($A_{\parallel}$) and perpendicular to it ($A_{\perp}$), as shown in Figure S3. The order parameter was calculated by $S = \frac{A_{\parallel}(\lambda^*) - A_{\perp}(\lambda^*)}{A_{\parallel}(\lambda^*) + 2A_{\perp}(\lambda^*)}$, where λ^* is the absorption peak of the dye. The order parameter was found to be $S = 0.407 \pm 0.026$.

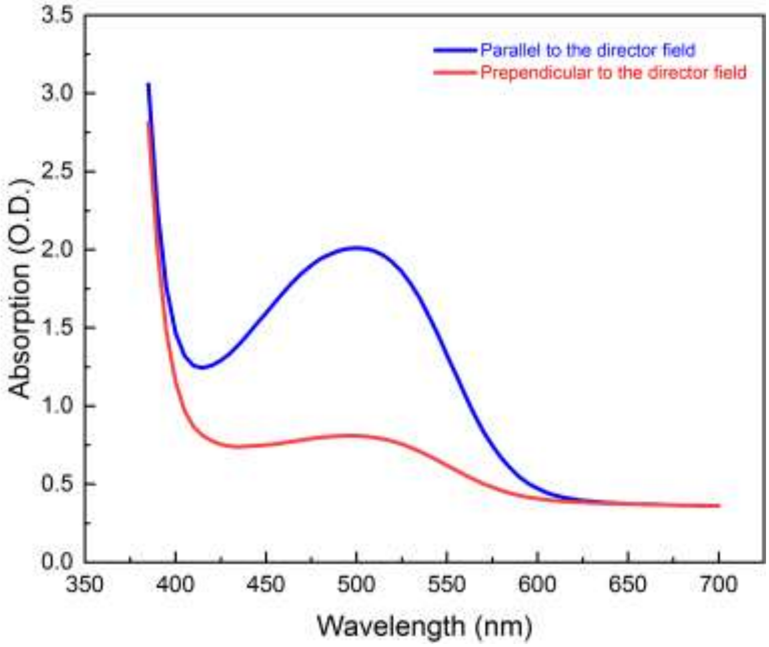

Figure S3. Absorption spectra obtained by the illumination of an LCN sample with a linearly polarized broadband light parallel and perpendicular to its optical axis.

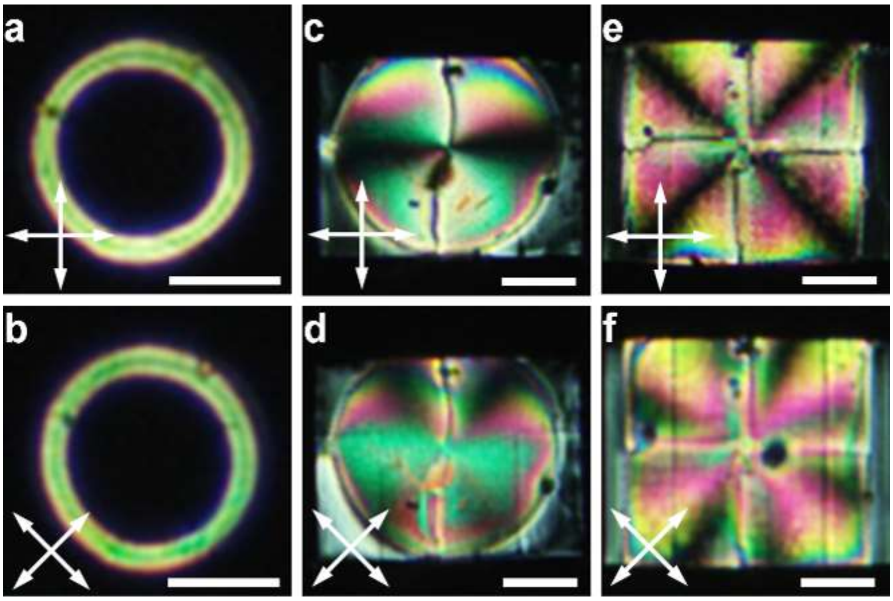

Figure S4. 3D LCN microstructures with 3D designable director field. (a, b) POM images of ring with twisting director field. (c, d) POM images of film with +1 topological defect pattern on the bottom and uniform alignment on top substrate. (e, f) POM images of film with +2 topological defect pattern on the bottom and uniform alignment on top substrate. Double-sided arrows exhibit the optical axis of polarizers. Scale bars are 50 μm in all images.

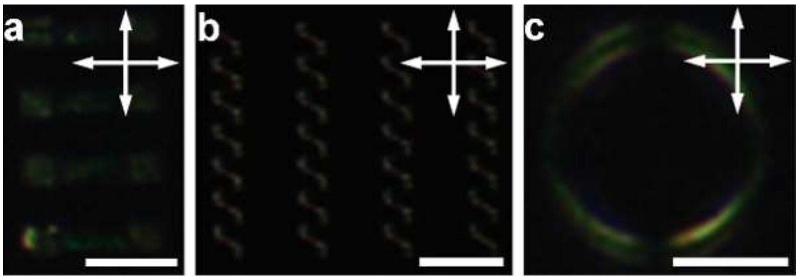

Figure S5. 3D LCN microstructures with uniform alignment. (a-c) POM image of film at a different height (a), coils (b), and ring (c). Double-sided arrows exhibit the optical axis of polarizers. Scale bars are 50 µm in all images.

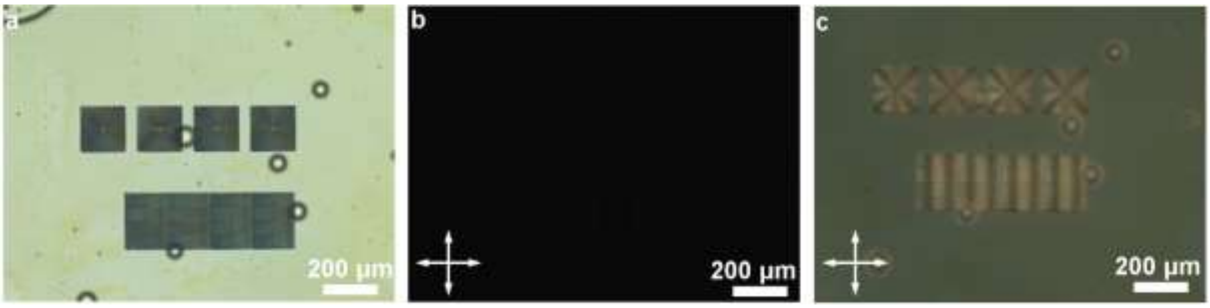

Figure S6. Grating effect from micro-channels. (a) Bright field image of sinusoidal patterns and +2 topological defect patterns. (b) POM image of sinusoidal patterns and +2 topological defect patterns. (c) 100 times exposure of POM image in (b). Note that (a) and (b) have same exposure with images in Figure 1.

S3. Swelling Experiments

The fabricated LCN micro-structure attached to a glass substrate was fixed under an optical microscope. A small droplet of DMF was dispensed on to the glass substrate to flood the LCN microstructure, which then detached from the glass substrate and swelled into a new shape. We defined the swelling ratio as the ratio between the length of the swollen structure and that of the innate structure. The swelling ratio along certain directions of our LCN microstructures are presented in Table S1. We also recorded the shape transformation processes as shown in Movie S1-S3. From these movies, we observed that the whole process of swelling took place rapidly, ca. 1-3 seconds. We think that the very small size of our LCN structures facilitates the fast sorption process and solvent diffusion.

We conducted a complementary set of experiments to investigate the effect of laser scanning direction and the type of photo-polymerization (i.s. flood exposure vs. two-photon polymerization) on the overall polymerization extent. In the first set of experiments, we fabricated two groups of thin films from LCNs with planar molecular alignment. The first group was swollen in DMF as fabricated. The second group, however, was exposed to the flood exposure $1\,J/cm^2$ for 8 minutes before the swelling with DMF. In order to investigate the effect of polymerization direction (scanning direction) we crosslinked similar thin films of LCN with a planar director field but with three different scanning directions with respect to the molecular alignment. Swelling experiments on all samples resulted in very similar equilibrium swelling ratios. These results, which are shown in Figure S8, indicate that our

LCNs are fully polymerized regardless of the direction of laser scanning in two-photon polymerization and the shape of LCN microstructures.

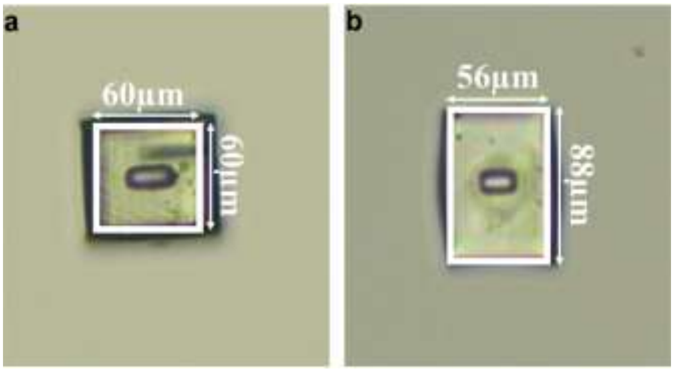

Figure S7. Shape transformation of the LCN cube with uniform alignment. (a) Optical microscope image of the fabricated cube with 60 μm side length. (b) Optical microscope image of the cube after swelled in DMF. A rectangular hole is fabricated on top of the LCN structure to show the orientation of the director field.

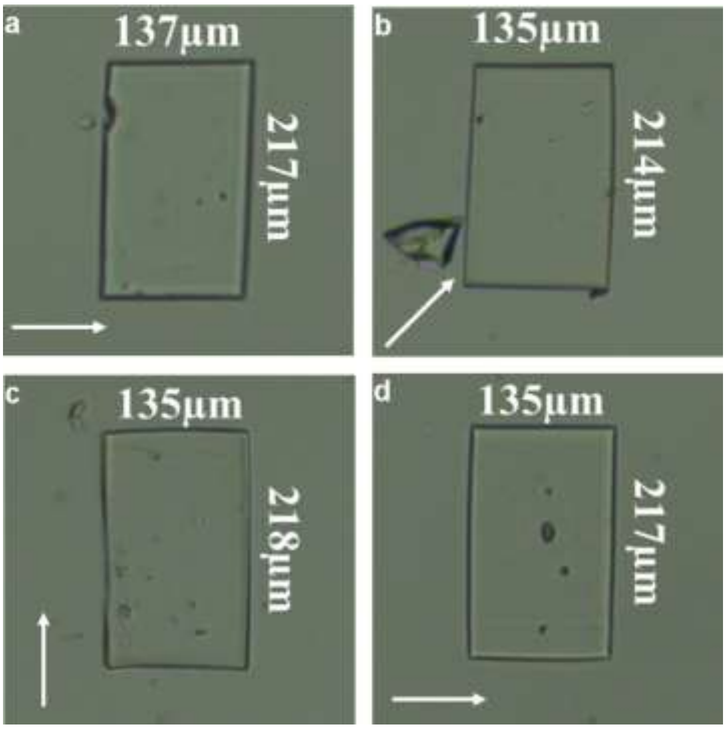

Figure S8. Swelling of square LCN films fabricated with different conditions. (a) Swelling of a square film with 150×150×10 μm size with 0° laser scanning direction. (b) Swelling of a square film with 150×150×10 μm size with 45° laser scanning direction. (c) Swelling of a square film with 150×150×10 μm size with 90° laser scanning direction. (d) Swelling of a square film in (a) with extra 1 J/cm^2 UV flood exposure after laser scanning. White arrows in each figure show laser scanning directions.

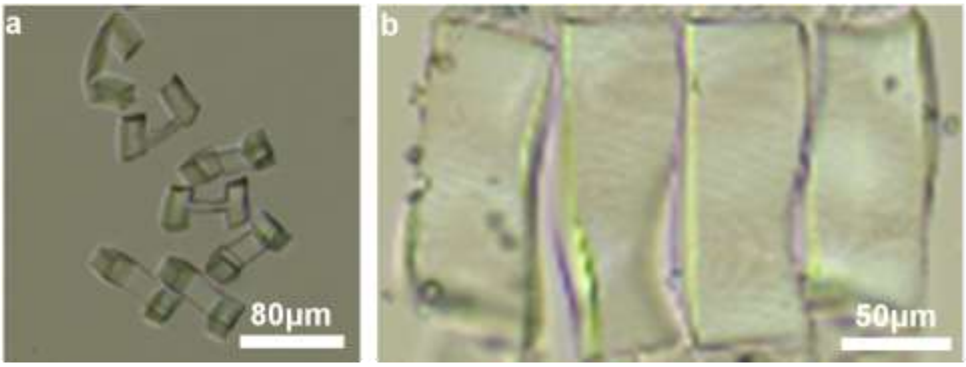

Figure S9. Shape-change of films. (a) Swell of suspended films in Figure 4(a). (b) Swelling of films with sinusoidal alignment in Figure 3(a).

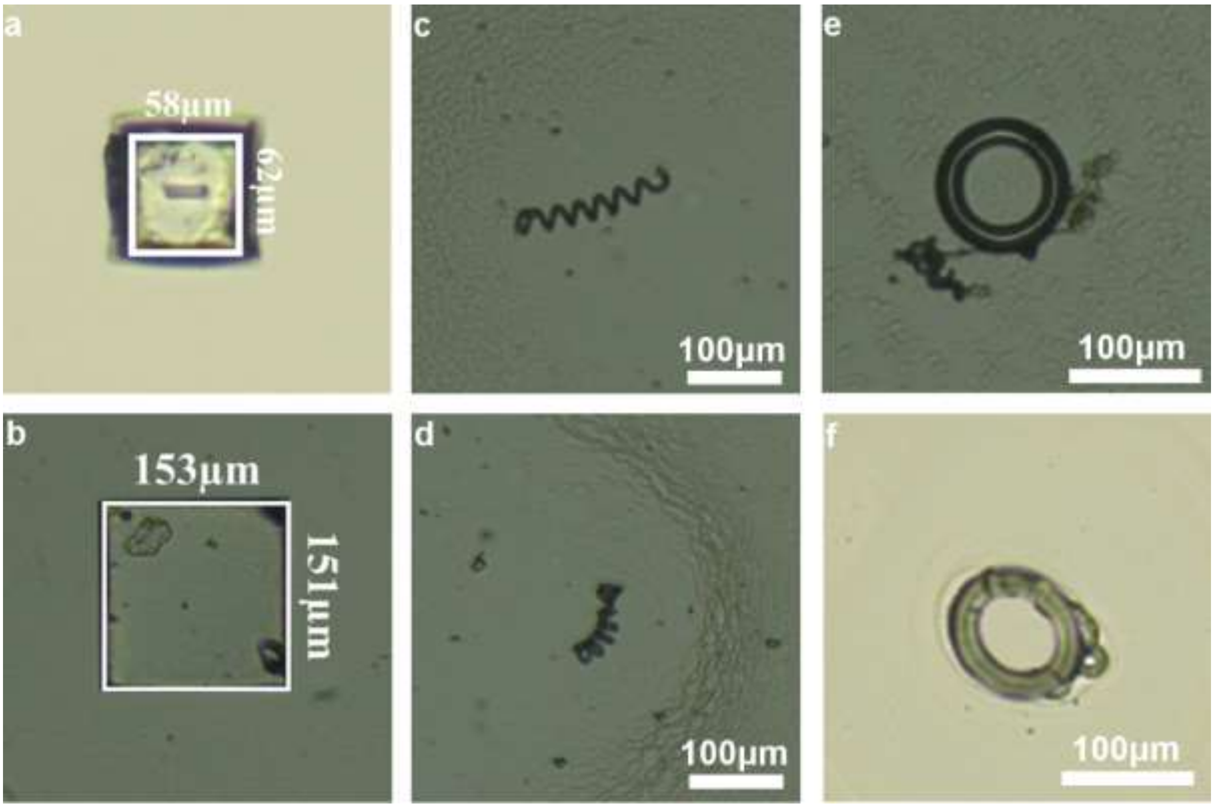

Figure S10. Shape-change reversibility. (a) The cube in Figure S7 after the evaporation of DMF. (b) The square film in Figure S8 after evaporation of DMF. (c) The coil in Figure 5a(i) after evaporation of DMF. (d) The coil in Figure 5a(iv) after evaporation of DMF. (e) The ring in Figure 5b(i) after evaporation of DMF. (f) The ring in Figure 5b(iii) after evaporation of DMF.

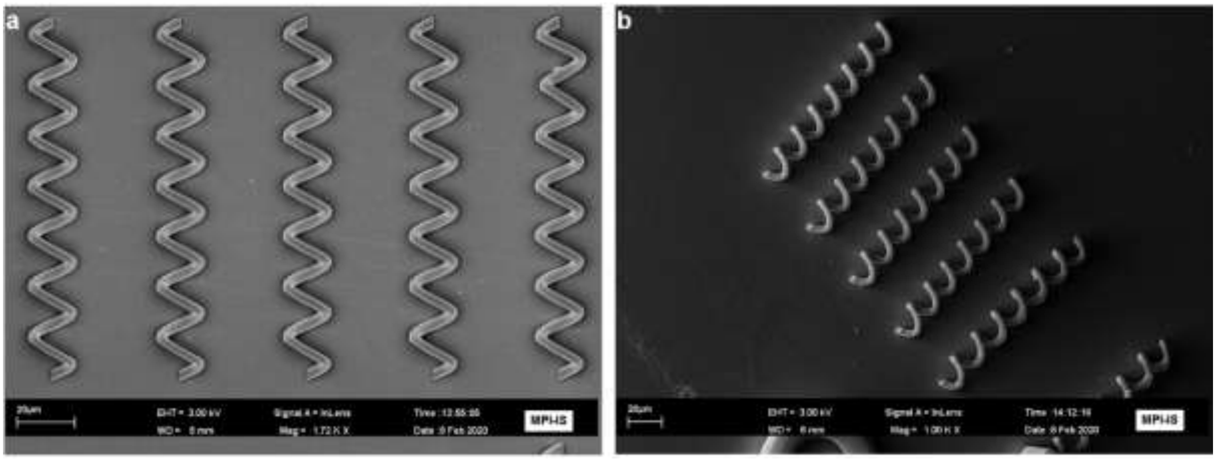

Figure S11. SEM images of LCN coils: (a) Top-view of the coils; (b) Tilted view of the coils with the tilt angle of 35°.

Table S1. Swelling of films, coils, and rings with different director fields with DMF.

Original shape	Alignment	Swelled shape	x-axis ratio	y-axis ratio
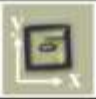	Uniform along x-axis	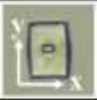	0.93	1.47
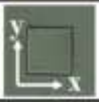	Uniform along x-axis	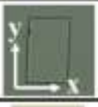	0.92	1.45
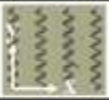	Fig. 5a(i)	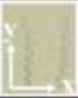	1.12	1.37
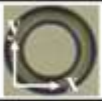	Fig. 5b(i)	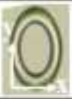	0.86	1.37
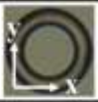	Fig. 5b(iv)	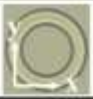	1.37	1.43

Supporting Movies

Movie S1. Swelling of a square film with uniform alignment. The movie is shown with real-time speed.

Movie S2. Swelling of coils with different encoded director fields. The movie is shown with 0.5X speed.

Movie S3. Swelling of rings with different encoded director fields. The movie is shown with real-time speed.
